# Social Media Messaging to Reduce HIV‐Related Stigma Among Young Adults in Peru: A Randomized Controlled Study

**DOI:** 10.1002/jia2.70168

**Published:** 2026-07-25

**Authors:** Queen Balina, Alyson Nunez, Milagros Wong, Marguerite Curtis, Kristin Kosyluk, Jerome T. Galea, Renato Errea, Molly F. Franke

**Affiliations:** ^1^ Faculty of Arts and Sciences Harvard University Boston Massachusetts USA; ^2^ Department of Global Health and Social Medicine Harvard Medical School Boston Massachusetts USA; ^3^ Socios En Salud Sucursal Perú Lima Perú; ^4^ Department of Mental Health Law and Policy University of South Florida Tampa Florida USA; ^5^ School of Social Work University of South Florida Tampa Florida USA; ^6^ Department of Epidemiology T.H. Chan School of Public Health Boston Massachusetts USA

**Keywords:** Bogardus scale, campaign, digital intervention, influencer, intervention, social distance

## Abstract

**Introduction:**

HIV‐related stigma undermines prevention and treatment efforts, particularly among young adults. Social media offers a scalable platform for stigma‐reduction efforts; however, empirical evidence of its effectiveness remains limited. Social media influencers may enhance the effectiveness of stigma reduction messages delivered via social media.

**Methods:**

From November 2024 to January 2025, we conducted a randomized controlled study in Lima, Peru, among young adults aged 18–29 years to evaluate the impact of brief exposure to tailored social media content designed to reduce HIV‐related stigma. Content featured widely recognized Peruvian influencers or young adult residents filmed in recognizable places in Lima. Participants were randomized to a simulated social media feed containing one of four HIV stigma‐reduction videos, including one featuring a top Peruvian influencer, or a control video. Stigma was evaluated using the Bogardus social distance scale, scored using standard and intensity‐based methods.

**Results:**

Five hundred and sixty‐seven persons participated (53% male, median age: 21 years). Median social distance pre‐randomization score was 1 in controls (fifth–95th percentile: 1–7), 1 in Arm 1 (fifth–95th: 1–7), 1 in Arm 2 (fifth–95th: 1–6), 1 in Arm 3 (fifth–95th: 1–5) and 1 in Arm 4 (fifth–95th: 1–7). Comparisons of pre‐ and post‐changes by study arm supported modest intervention effects. Controls showed a median change of 0 (fifth–95th: –1–2). Arm 1, featuring influencer‐generated content, showed a median change of 0 (fifth–95th: –6–0), with a Wilcoxon rank‐sum test indicating a statistically significant difference from controls (*p* = 0.001). Arm 2 showed a median change of 0 (fifth–95th: –1–0, *p* = 0.08). Arm 3 showed a median change of 0 (fifth–95th: –2–0, *p* = 0.01). Arm 4 showed a median change of 0 (fifth–95th: –3–0, *p* = 0.01).

**Conclusions:**

Brief exposure to culturally tailored social media content led to modest reductions in HIV‐related stigma among young adults in Peru immediately after viewing. Findings reinforce the potential role of social media and influencers in HIV stigma reduction efforts. Future research should examine whether social media interventions can reduce stigma among those with the most stigmatizing beliefs and identify dosing and delivery strategies that achieve durable reductions in stigma.

## 1 Introduction

HIV‐related stigma undermines prevention and treatment efforts globally and remains a barrier to achieving UNAIDS 95‐95‐95 targets [1, 2]. This stigma shapes public perceptions of people living with HIV (PLWHIV) and contributes to delays in healthcare utilization, reduced engagement in care and social exclusion among PLWHIV or at risk for HIV acquisition [3, 4, 5, 6]. Adolescents and young adults, a group that disproportionately experiences unfavourable HIV‐treatment outcomes, may be especially vulnerable to the harmful effects of stigma due to developmental and life‐stage factors [7, 8, 9].

Understanding how stigma operates is essential for developing effective interventions to mitigate its consequences. The Health Stigma and Discrimination Framework, applied across a range of health conditions, conceptualizes stigma as a process in which drivers (e.g. lack of information) and facilitators (e.g. key power groups) reinforce stereotypes, prejudice and discrimination, resulting in adverse health and social outcomes [10]. Building on this framework, interventions that target key drivers and facilitators, such as education‐based approaches that correct misinformation and contact‐based approaches that promote exposure to lived experiences and foster empathy, have been shown to reduce stigma [11, 12], including HIV‐related stigma [13, 14, 15]. In the context of HIV, storytelling (sharing of lived experience), community‐informed messaging and engagement of peers or popular opinion leaders are key strategies underpinning information‐ and contact‐based approaches [16, 17, 18, 19]. Stigma reduction interventions have typically targeted PLWHIV and other priority groups (e.g. healthcare workers, key populations at high risk for acquiring HIV), rather than the broader public [13, 14, 20, 21].

As internet access and social media use continue to expand globally, digital interventions have emerged as scalable approaches for broad dissemination of HIV‐related information, particularly to young people [22]. Studies have demonstrated that tailored digital content is effective at improving HIV knowledge, awareness of prevention and care services, and uptake of HIV testing over the short‐term [23, 24, 25, 26, 27, 28]. Although explicitly addressing stigma as part of digital interventions to increase uptake and engagement in HIV‐related services would likely accelerate progress and promote sustained success, few studies have examined HIV‐related stigma as an outcome or potential mediator. Whether innovative digital strategies that explicitly target stigma can further help bridge treatment and care gaps remains an open question.

One such strategy involves partnering with influencers (i.e. social media personalities with large, engaged followings) who can reduce stigma and prejudice by serving as trusted messengers, fostering parasocial relationships (non‐reciprocal socio‐emotional connections with media figures) and engaging their audience [29, 30]. Exposure to influencer content within users’ everyday online environments has been shown to shape users’ beliefs [31]. By modelling acceptance and empathy and clarifying HIV‐related misconceptions, influencers may help reduce stigma towards marginalized groups, including PLWHIV. However, limited empirical research has examined whether influencer‐created content can measurably reduce HIV‐related stigma, particularly among young adults.

Peru is a Latin American country with a concentrated HIV epidemic. While the HIV prevalence in the general adult population is less than 1% [32], it is higher among key populations in Lima, the nation's capital. Among men who have sex with men, the estimated HIV prevalence is 10% and, among transgender women, it is as high as 30% [32]. In 2024, 44% of new diagnoses occurred in young adults aged 18−29 [33]. Stigma is a substantial barrier to care in Peru, and public attitudes and disclosure concerns are associated with disengagement from HIV care [34]. Social media use is common in Peru, with over 70% of adults aged 18−35 reporting daily use [35], creating an opportunity to engage young people who might otherwise be difficult to reach.

In this study, we examined whether brief, culturally tailored social media messaging, including influencer‐created content, could reduce HIV‐related stigma among young adults in Lima, Peru. We compared exposure to the intervention social media content to routine non‐health‐related content to assess short‐term effects on social distancing beliefs towards PLWHIV. By conducting this work in Peru, we aimed to address a key knowledge gap regarding stigma‐reduction strategies implemented in Latin America [14].

## 2 Methods

### 2.1 Study Design

We evaluated whether brief exposure to social media content targeting HIV‐related stigma led to reductions in stigma among young adults in Lima. Specifically, we employed a randomized controlled, pre‐post intervention study design to assess the impact of selected youth‐friendly, culturally tailored social media content on HIV‐related stigma, as compared to routine social media consumption. Intervention content was compared to routine social media consumption to create a pragmatic control similar to what young adults may be expected to encounter online.

### 2.2 Study Population and Recruitment

Individuals aged 18−29 were recruited in person from a large, private university in southern Lima and from urban public spaces with high pedestrian traffic, allowing for engagement with a heterogeneous young adult population. To ensure that populations disproportionately affected by HIV were represented in the sample, we also recruited participants from spaces frequented by young men who have sex with men and transgender women. Eligibility criteria included self‐reported social media use at least three times per week and the ability to provide verbal informed consent. HIV status was neither solicited as part of enrolment procedures nor used as an inclusion or exclusion criterion. Recruitment occurred through direct invitations to passersby. Study personnel, wearing uniforms identifying them as researchers working for a local NGO, enrolled young people during the afternoon and evenings, Monday through Saturday.

### 2.3 Content Development

The content we tested was developed as part of a community‐engaged social media campaign (nicknamed “DiME,” Spanish for “tell me”) designed to reduce HIV‐related stigma among young people with or without HIV [36]. Campaign content was in video format and developed based on formative work with young PLWHIV and other stakeholders [18]. Content drew on education‐ and contact‐based approaches to dispel stereotypes, normalize conversations about HIV and promote empathy; a detailed description of content creation is reported elsewhere [36]. To ensure youth‐friendliness and cultural relevance, content featured widely recognized Peruvian influencers or young adult residents of Lima filmed in recognizable places throughout the city. Content incorporated local slang and social media trends. All campaign social media content was approved by a community advisory group of 15 PLWHIV aged 19−37, four of whom were activists. The advisory group reviewed scripts and first‐draft videos, provided feedback and ensured that final versions adequately communicated messages.

**FIGURE 1:**
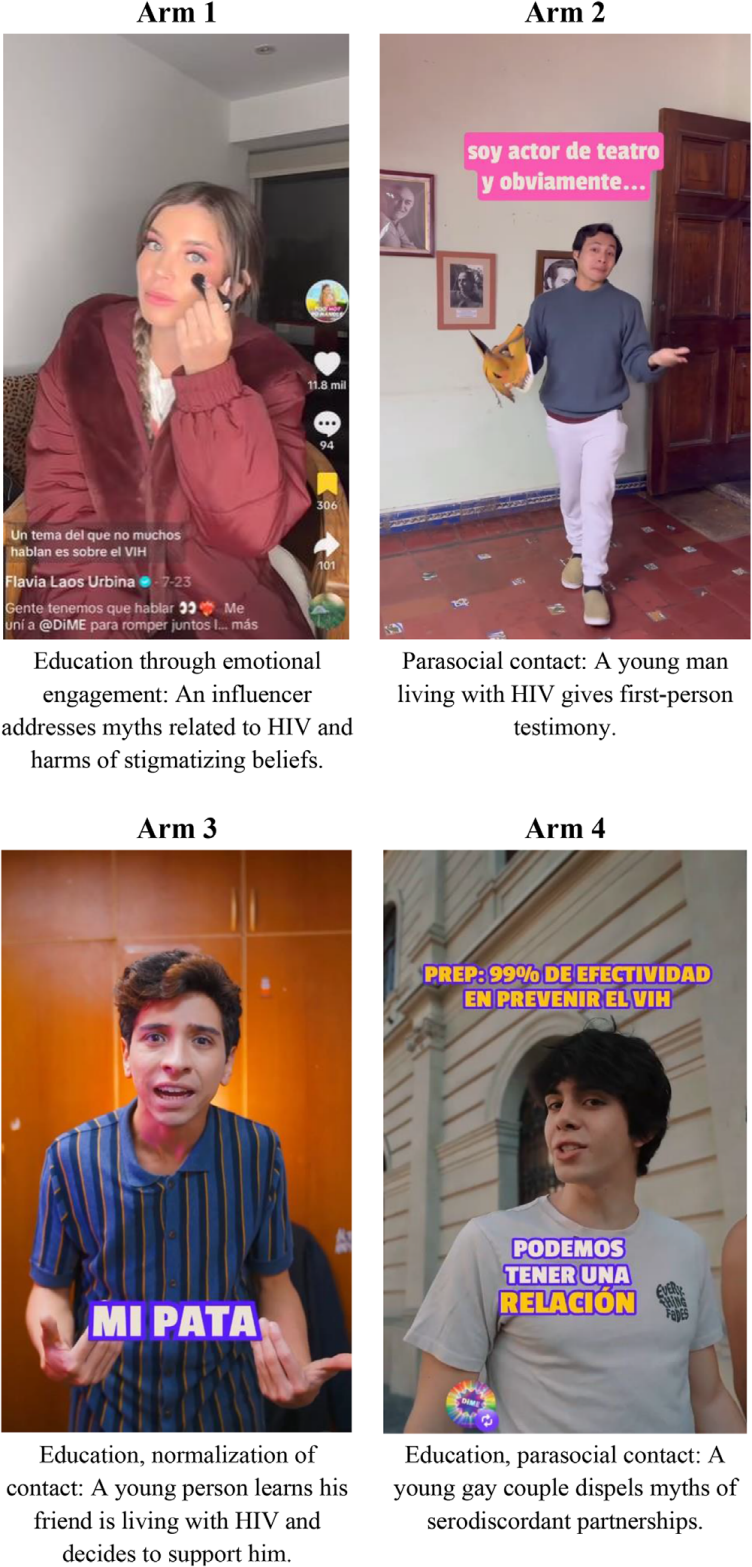
Description of intervention content. The authors have received consent to publish all images included.

Four pieces of campaign content were evaluated in this study: three produced by a social marketing company in collaboration with the research team and one created by an influencer. These pieces were selected for their high engagement during the campaign and a goal of testing different types of content (e.g. vignette‐based, testimony and influencer‐produced). Selected intervention content and communication mechanisms are shown in Figure 1.

### 2.4 Study Procedures

Participants were provided a tablet to complete a pre‐intervention survey. After the survey, they advanced to the next screen, where the database randomly assigned them to one of five simulated social media feeds, each containing three pieces of content. Randomization was implemented via the REDCap study database, using a blocked allocation sequence, with random block sizes. A U.S.‐based investigator created the sequence; all others were blinded to allocation.

The total duration of each simulated feed was 1.5−3 minutes. In each feed, the first and third videos featured non‐health‐related content popular in Peru at the time of the campaign (e.g. a TV show clip, a group dance or local influencer content). The second video in each feed was either one of the four HIV‐related pieces or control content similar to the first and third videos. After watching the social media feed, participants advanced to the next screen to complete the post‐intervention survey. Total participation time was approximately 10 minutes. Participants received a bag containing three condoms, candy and DiME campaign stickers as a token of appreciation. No direct or indirect identifiers were collected for study purposes, but participants could separately provide their contact information to be entered into a raffle to win a laptop.

### 2.5 Data Collection

The pre‐randomization survey included socio‐demographic characteristics (i.e. age, sex, gender, sexual orientation, educational attainment) and data related to HIV stigma, the primary outcome. HIV‐related stigma was assessed using a modified Bogardus Social Distance Scale. The original Bogardus scale, designed to measure social distance towards ethnic groups [37], has been adapted for other stigmatized conditions, including mental illness [38] and COVID‐19 [39]. In this adaptation, participants were presented with a narrative of a fictional character, “Juan,” described as “A young man who is smart and talented, devoted to his family and friends, and widely viewed as kind and responsible. Juan was recently diagnosed with HIV and is now receiving treatment.” Respondents rated their willingness to interact with Juan across six relational contexts (i.e. if he were a foreigner visiting Peru, a Peruvian citizen, a coworker, a neighbour, a close friend or a family member) on a 4‐point Likert scale, ranging from 1 (“definitely willing”) to 4 (“unwilling”), with higher scores indicating greater social distance. We removed the most distant item (“would exclude from entering my country”) from the original scale because, during pilot testing, participants could not clearly distinguish it from the next two furthest contexts (accepting as a foreign visitor or as a citizen), and its reversed wording caused confusion. Data collection took place over 2 months, from November 2024 to January 2025.

### 2.6 Statistical Analyses

In primary analyses, we calculated the Bogardus score by dichotomizing responses: scores of 1 or 2 were coded as “willing,” and scores of 3 or 4 were coded as “unwilling.” This approach aligns with the original proposed scoring for the Bogardus scale [37]. Participants were assigned a value corresponding to the first question to which they responded “unwilling.” Higher values correspond to relational contexts further from one's personal environment (i.e. a higher value indicates a greater relative social distance). For example, if a participant's first unwilling response was to the first item on the scale (i.e. “How willing would you be to accept Juan as a foreigner visiting Peru?”), they were assigned a score of 6. If their first unwilling response was to the second item (i.e. “How willing would you be to accept Juan as a citizen in Peru?”), they received a score of 5. A first unwilling response at the third item corresponded to a score of 4, at the fourth item a score of 3, at the fifth item a score of 2 and at the sixth item a score of 1.

In secondary analyses, we calculated the Bogardus Intensity Score (iScore), introduced by Mather and colleagues [40]. The iScore is calculated by multiplying the Likert response (1 through 4) for each question by its corresponding position on the Bogardus Scale (6 through 1), in reverse order, and summing the products. Possible iScores ranged from 21 (lower social distance) to 84 (higher social distance). For example, the Likert response to the first survey question is multiplied by 6, the second by 5 and so on, yielding a more nuanced index of respondents’ affective positioning.

We calculated changes in Bogardus social distance scores for each participant by subtracting respondents’ initial scores from their final scores after watching the simulated social media feed. We compared these changes between the intervention arms and the control arm using Wilcoxon rank‐sum tests (i.e. a two‐timepoint difference‐in‐differences comparison). Statistical analyses were conducted using SAS, and statistical significance was defined as *p* < 0.05.

### 2.7 Sample Size Calculation

We planned for a sample size of 50 participants per arm (i.e. 50 individuals exposed to control content and 50 individuals exposed to each intervention content evaluated). With a Type 1 error rate of 0.05, this sample size would have afforded 80% power to detect change differences of at least 0.57 under original Bogardus scoring (assuming a standard deviation of 1) and 2.8 using Bogardus iScores (assuming a standard deviation of 5). Our final sample size included more than twice this number of participants for each comparison.

### 2.8 Ethical Considerations

The study was reviewed and exempted under Common Rule Category 3 covering benign behavioural interventions in adults by the Harvard Longwood Campus Institutional Review Board and the Vía Libre Institutional Committee of Bioethics in Peru.

## 3 Results

### 3.1 Participant Characteristics

**FIGURE 2:**
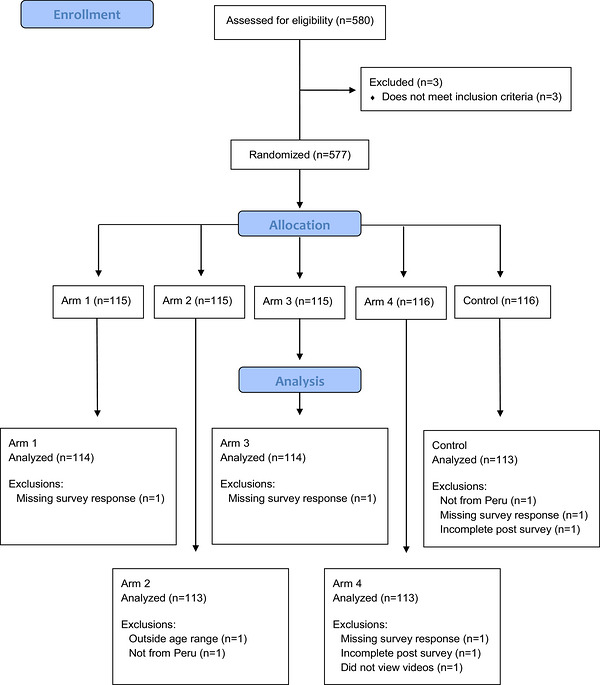
CONSORT (Consolidated Standards of Reporting Trials) diagram.

Of 580 participants randomized, we excluded 13 (2%): four reported an age outside of the eligibility criteria, and nine were post hoc exclusions due to missing a pre‐ or post‐response to the Bogardus scale, not residing in Peru, not completing post‐survey questions and not watching the assigned video content (Figure 2).

The final analytic sample included 567 participants. The median age was 21 years (IQR: 19–24). Most participants were born in Peru (97%), and half had completed or were enrolled in higher education (50%). Baseline demographic characteristics were generally balanced across the five arms (Table 1). Twenty‐three percent of participants reported knowing someone living with HIV. Thirty‐nine percent of participants were enrolled at their university; the remainder were enrolled in public spaces (e.g. a civic centre, a main street, a shopping district) in downtown Lima.

**TABLE 1:** Participant characteristics by study arm.

	Control	Arm 1	Arm 2	Arm 3	Arm 4
Characteristics	*n* = 113 (%)	*n* = 114 (%)	*n* = 113 (%)	*n* = 114 (%)	*n* = 113 (%)
Median age (IQR)	21 (19−23)	21 (19−24)	21 (19−24)	21 (19−25)	21 (19−24)
Age					
18–23	86 (76.1)	81 (71.0)	80 (70.8)	77 (67.5)	82 (72.6)
24–29	27 (23.9)	33 (29.0)	33 (29.2)	37 (32.5)	31 (27.4)
Birth country					
Peru	110 (97.3)	113 (99.1)	107 (94.7)	107 (93.9)	112 (99.1)
Other	3 (2.7)	1 (0.9)	6 (5.3)	7 (6.1)	1 (0.9)
Education					
Less than secondary	0	0	0	1 (0.9)	0
Secondary	38 (33.6)	39 (34.2)	36 (31.9)	50 (43.9)	42 (37.5)
Trade school	15 (13.3)	14 (12.3)	19 (16.8)	12 (10.5)	17 (15.2)
University or higher	60 (53.1)	61 (53.5)	58 (51.3)	51 (44.7)	53 (47.3)
Sex (assigned at birth)					
Female	52 (46.0)	56 (49.1)	50 (44.2)	51 (44.7)	57 (50.4)
Male	61 (54.0)	57 (50.0)	63 (55.8)	63 (55.3)	56 (49.6)
Intersex	0	1 (0.9)	0	0	0
Gender (identity)					
Woman (cisgender)	51 (45.1)	56 (49.1)	48 (42.5)	49 (43.0)	58 (51.3)
Man (cisgender)	57 (50.4)	57 (50.0)	62 (54.9)	62 (54.4)	54 (47.8)
Transgender woman	3 (2.7)	0	0	0	0
Transgender man	0	1 (0.9)	3 (2.7)	1 (0.9)	1 (0.9)
Genderfluid	1 (0.9)	0	0	0	0
Nonbinary	0	0	0	1 (0.9)	0
Other	1 (0.9)	0	0	1 (0.9)	0
Sexual orientation					
Heterosexual	91 (81.3)	90 (79.0)	89 (78.8)	93 (81.6)	87 (77.0)
Homosexual	6 (5.4)	9 (7.9)	14 (12.4)	6 (5.3)	10 (8.9)
Bisexual	8 (7.1)	12 (10.5)	6 (5.3)	12 (10.5)	12 (10.6)
Pansexual	3 (2.7)	2 (1.8)	1 (0.9)	2 (1.8)	2 (1.8)
Other	4 (3.6)	1 (0.9)	3 (2.7)	1 (0.9)	2 (1.8)
Knows someone living with HIV (*N* = 549)	24 (21.8)	18 (16.5)	29 (26.4)	33 (29.7)	24 (22.0)
Family or friend	22 (91.7)	14 (77.8)	20 (69.0)	26 (78.8)	18 (75.0)
Acquaintance	2 (8.3)	4 (22.2)	9 (31.0)	7 (21.2)	6 (25.0)

### 3.2 Primary Analyses

Baseline Bogardus scores were comparable across all five study arms (Table 2). The median social distance pre‐randomization score was 1 in the control arm (fifth−95th percentile: [1−7]), 1 in Arm 1 (fifth–95th percentile: [1–7]), 1 in Arm 2 (fifth–95th percentile: [1–6]), 1 in Arm 3 (fifth–95th percentile: [1–5]) and 1 in Arm 4 (fifth–95th percentile: [1–7]). Pre‐ and post‐exposure response frequencies for all Bogardus scale items are presented in Tables S1–S3.

**TABLE 2:** Pre‐randomization Bogardus scale scores and iScores, by study arm.

	Bogardus score median [fifth−95th percentile]	Bogardus iScore median [fifth−95th percentile]
Control	1 [1−7]	27 [21−54]
Arm 1	1 [1−7]	26 [21−55]
Arm 2	1 [1−6]	24 [21−47]
Arm 3	1 [1−5]	24 [21−45]
Arm 4	1 [1−7]	28 [21−58]

**FIGURE 3:**
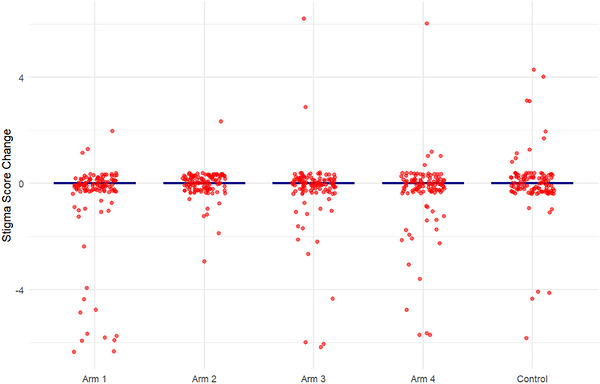
Distribution of changes in Bogardus scale scores, by study arm. Bars indicate median change in score (0) for each arm. Wilcoxon rank sum *p*‐values for changes in stigma score in intervention arms, relative to the control arm, were 0.001 for Arm 1, 0.08 for Arm 2, 0.01 for Arm 3 and 0.01 for Arm 4.

The control arm demonstrated little change in Bogardus score between pre‐ and post‐survey assessments (median change = 0; fifth–95th percentile: [−1–2]; Table 3 and Figure 3). Although the majority of intervention participants also did not experience a change between the pre‐ and post‐surveys (median change = 0 in all arms), participants assigned to view intervention content were more likely to experience reductions in stigma, and these tended to be modest (Table 3 and Figure 3). The largest reduction relative to the control arm was observed in Arm 1, which featured influencer‐produced content (Bogardus score change fifth–95th percentile: [−6–0]; Wilcoxon rank‐sum test for change compared with the control arm *p* = 0.001). Smaller but statistically significant reductions were observed in Arm 3 (fifth–95th percentile: [−2–0]; *p* = 0.01) and Arm 4 (fifth–95th percentile: [−3–0]; *p* = 0.01). Arm 2 demonstrated the smallest magnitude of change relative to the control arm, which was not statistically significant (fifth–95th percentile: [−1–0]; *p* = 0.08) (Figure 3 and Table S4).

**TABLE 3:** Change in Bogardus scale scores, by study arm.

	Decrease in score	No change in score	Increase in score
Arm 1	20 (17.5%)	91 (79.8%)	3 (2.6%)
Arm 2	7 (6.2%)	105 (92.9%)	1 (0.9%)
Arm 3	15 (13.2%)	97 (85.1%)	2 (1.8%)
Arm 4	18 (15.9%)	90 (79.6%)	5 (4.4%)
Control	7 (6.2%)	96 (85.0%)	10 (8.8%)

**FIGURE 4:**
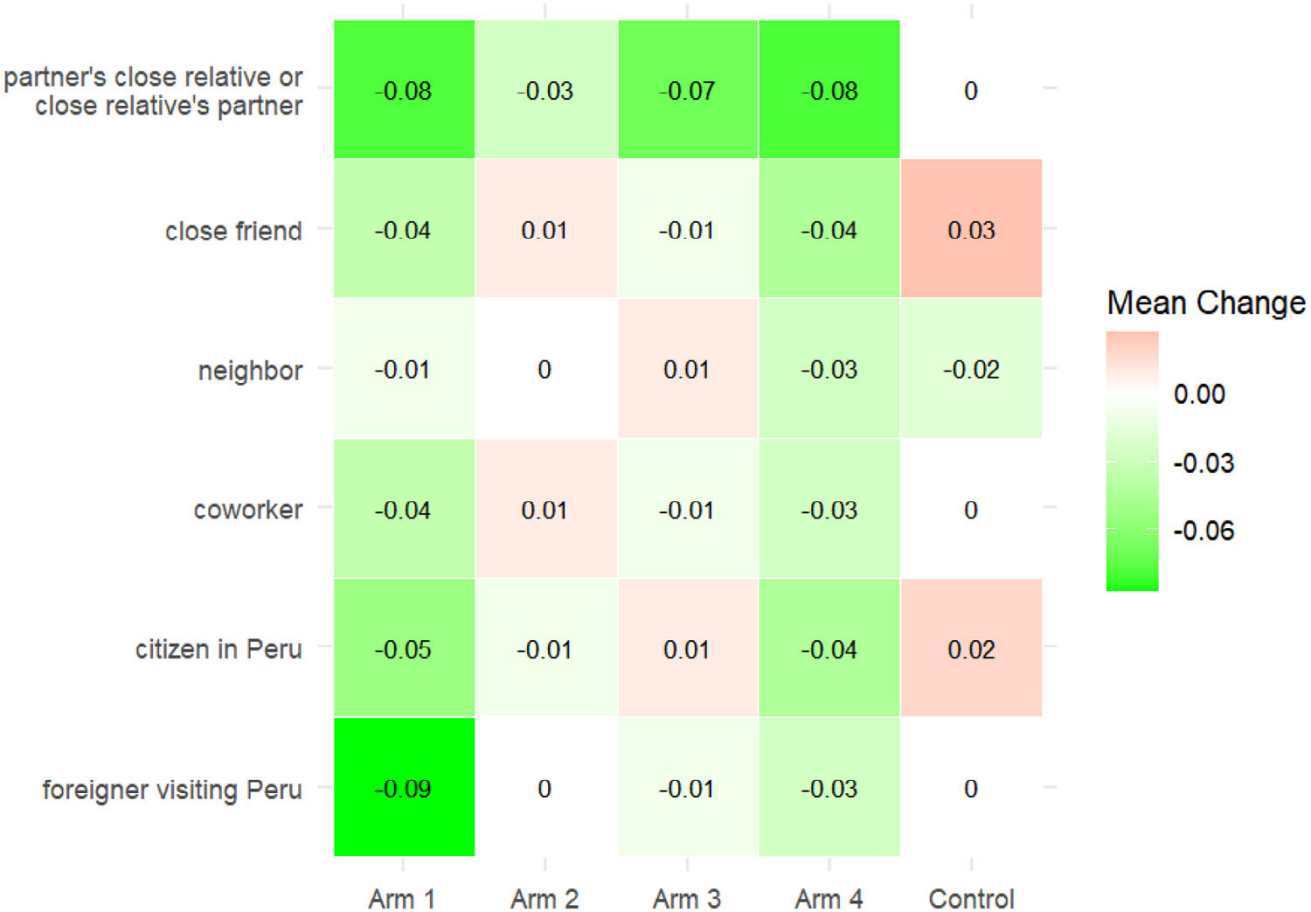
Heatmap of mean within‐person change in Bogardus stigma score by arm and question. Each question asks “*How willing would you be to accept Juan as a …*”.

Across study arms, the survey item assessing willingness to accept Juan as “a close relative of your partner or as a partner of your family member” demonstrated the greatest shift in responses following content exposure (Figure 4).

### 3.3 Secondary Analyses

**FIGURE 5:**
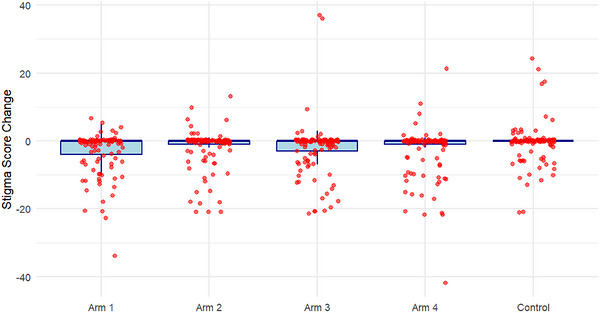
Distribution of changes in Bogardus scale iScore, by study arm. Bars indicate median change in score (0) for each arm. Shaded blocks correspond to the interquartile range. Wilcoxon rank sum *p*‐values for changes in stigma score in intervention arms, relative to the control arm, were 0.01 for Arm 1, 0.16 for Arm 2, 0.01 for Arm 3 and 0.05 for Arm 4.

Findings from secondary analyses were similar to results from primary analyses. Baseline median iScores were comparable across all study arms (Table 2). Median change across all arms was 0; however, those randomized to view intervention content experienced a greater overall reduction in iScores than controls (Figure 5), and these differences were statistically significant in Arms 1, 3 and 4 (Table S5).

## 4 Discussion

Our findings demonstrate the potential of even brief exposure to youth‐friendly, culturally tailored social media content for reducing HIV‐related stigma among young adults. Participants exposed to intervention content more frequently demonstrated decreases in HIV‐related stigma: modest, statistically significant reductions were observed in three of the four intervention arms. While the intervention content varied, it consistently emphasized authenticity, relatability and non‐stigmatizing portrayals of PLWHIV, reinforcing the importance of these features in effective digital stigma‐reduction messaging. The influencer‐generated content, which centred on empathy and personal connection, demonstrated the greatest reduction in stigma; however, this may be a chance finding, as the study did not test differences between intervention arms. Taken together, these findings suggest that social media content, including content featuring influencers, has the potential to reduce stigmatizing beliefs and could be considered as a wide‐reaching, low‐cost component of a broader stigma‐reduction strategy.

By studying social media content in a controlled environment simulating routine social media use among youth and measuring a stigma‐specific outcome, these findings extend a growing body of research on social media‐based interventions aimed at increasing uptake of HIV‐related services [27, 41, 42, 43, 44, 45, 46, 47]. Many prior studies describing the feasibility of HIV‐related social media interventions lack robust evaluations of effectiveness, perhaps in part due to challenges inherent to assessing exposure to a population‐based social media intervention [48]. Exceptions include the HOPE social media campaign in Peru, which led to significant increases in testing uptake and reductions in stigma‐related barriers [44], and the *IknowUshould2* campaign [43], which was associated with increased HIV and syphilis testing. Although explicitly addressing stigma as part of efforts to scale up engagement in HIV‐related care may accelerate progress towards this goal and facilitate long‐term engagement, few studies have measured stigma‐related outcomes, with a notable example being a crowdsourced intervention in Kazakhstan that led to lower HIV testing stigma among young people [49].

Of the four intervention pieces evaluated, the influencer‐created content, which was intended to elicit empathy and compassion, produced the most consistent reductions in stigma, though the intervention content was not formally compared to each other. Other studies have similarly identified the importance of influencers’ familiarity with the local realities, and their ability to lead with authentic, compassionate, “human”‐focused messaging [50, 51]. A 2023 HIV prevention campaign in the southern United States found greater engagement with HIV prevention messaging delivered by Black social media influencers than with paid advertisements alone [52]. The distinctive power of influencers, particularly those perceived as authentic and reliable, to shift norms, reduce stigma and drive health‐related behaviour change among young adults [12, 53], makes them potentially powerful catalysts for expanding the reach and penetration of stigma‐reduction messaging, particularly for populations that may otherwise be difficult to access with this information [30]. Working in partnership with influencers and ensuring adequate sensitization will minimize unintended negative effects, such as the dissemination of misinformation [30].

Cultural and emotional specificity are critical for effective messaging, and research has found that stigma‐reduction efforts often fall short when messages lack local relevance or fail to engage their audiences emotionally [10, 54]. Our content was intentionally designed to be culturally relevant and relatable to young adults by featuring Peruvian influencers and actors in intimate settings and using direct, casual language. This approach aligns with prior work highlighting the importance of emotionally salient, culturally grounded messaging for effectively engaging youth [55]. However, the video in Arm 2, which had the least pronounced reduction in stigma, may not have evoked sufficient emotion. The young man featured in that video leveraged a viral trend to communicate his testimony about living with HIV. The first five seconds consisted of a silent clip from a theatre performance, showing him disclosing his diagnosis to his family, before transitioning to scenes highlighting his experiences as an actor living with HIV. Careful attention to the subtitles was necessary to capture the video's message, which may have made it challenging for some viewers to follow.

Several limitations should be considered. Across all study arms, a majority of people did not experience any reduction in HIV‐related stigma, perhaps because HIV‐related stigma, as assessed by the social distancing scale, was relatively low. Although we employed a randomized design, participants assigned to view intervention content may have inferred the study objective, raising the possibility of social desirability bias. Stigma outcomes were assessed immediately post‐exposure, so the durability of these reductions remains unknown. Longitudinal data will be necessary to determine whether effects persist over time. The study targeted individuals 18−29 years in a major urban area; it is unknown whether these results generalize to older populations or rural communities. Finally, because each intervention arm differed in tone, visual style, messenger credibility and format, and the study was not powered to draw comparisons across arms, it was not possible to determine which content and/or attributes were most impactful for stigma reduction.

The growing body of literature supporting social media‐based stigma‐reduction strategies raises important questions for future research. For example, it is unknown if social media interventions can reach and impact opinions among people who hold more stigma and may be less likely, due to personal avoidance and/or algorithm‐based content curation, to be exposed to HIV‐related outreach. The long‐term impact of brief digital stigma‐reduction interventions and the ways in which “dosing” and delivery style enhance or sustain effectiveness should also be explored. Finally, additional work is needed to identify which elements of the content most strongly influence stigma reduction. Mixed‐method designs incorporating qualitative feedback could clarify mechanisms of impact and how various populations interpret different types of content.

## 5 Conclusions

In conclusion, this study found that brief exposure to targeted social media messages about HIV can reduce social distance among young adults immediately following the intervention. These findings suggest that culturally tailored and influencer‐driven content may have potential as an approach to addressing HIV‐related stigma in urban, resource‐limited settings with high social media engagement. Leveraging innovative approaches for stigma reduction will be critical to closing gaps in prevention and treatment and, ultimately, advancing global efforts to end the HIV epidemic.

## Author Contributions

*Conceptualization*: MFF, RE, JTG, KK and MW. *Data curation*: MC and MW. *Formal analysis*: QB and AN. *Funding acquisition*: MFF, RE, JTG and KK. *Investigation*: All authors. *Methodology*: MFF, RE, KK, MW and AN. *Project administration*: MW and MC. *Supervision*: MFF, RE, MW and AN. *Writing – original draft preparation*: QB and MFF. *Writing – review and editing*: All authors. All authors have read and approved the final manuscript.

## Funding

This work was funded entirely by the U.S. National Institutes of Health under award R01TW012394.

## Conflicts of Interest

The authors declare that they have no conflicts of interests.

## Disclaimer

The content is solely the responsibility of the authors and does not necessarily represent the official views of the National Institutes of Health.

## Supporting information




**Table S1**: Survey response frequency before exposure to social media content by study arm.
**Table S2**: Survey response frequency after exposure to social media content by study arm.
**Table S3**: Survey response frequency before and after exposure to social media content by study arm.
**Table S4**: Median Bogardus change by study arm (original scoring).
**Table S5**: Median Bogardus iScore change by study arm.

## Data Availability

The data that support the findings of this study are available from the corresponding author upon reasonable request.
