# Supplementary material for: Social Media Messaging to Reduce HIV‐Related Stigma Among Young Adults in Peru: A Randomized Controlled Study
Source: J Int AIDS Soc. 2026 Jul 25;29(Suppl 3):e70168. doi: 10.1002/jia2.70168 (PMC13401724; doi:10.1002/jia2.70168)
Supplement: Supplementary file 1 — Table S1: Survey response frequency before exposure to social media content by study arm. Table S2: Survey response frequency after exposure to social media content by study arm. Table S3: Survey response frequency before and after exposure to social media content by study arm. Table S4: Median Bogardus change by study arm (original scoring). Table S5: Median Bogardus iScore change by study arm. [file JIA2-29-e70168-s001.docx]

**Supplementary Material**

**Social Media Messaging to Reduce HIV-Related Stigma Among Young Adults in Peru: A Randomized Controlled Study**

**Authors:** Queen Balina^1^, Alyson Nunez^2^, Milagros Wong^3^, Marguerite Curtis^2^, Kristin Kosyluk^4^, Jerome T. Galea^5^, Renato Errea^3*^, Molly F. Franke^2,6*^

1. Faculty of Arts and Sciences, Harvard University
2. Department of Global Health and Social Medicine, Harvard Medical School
3. Socios En Salud Sucursal Perú, Lima, Perú
4. Department of Mental Health Law and Policy, University of South Florida, Tampa, Florida, USA
5. School of Social Work, University of South Florida
6. Department of Epidemiology, T.H. Chan School of Public Health

*Contributed equally

**Corresponding Author:**

Molly F. Franke

[Molly_franke@hms.harvard.edu](mailto:Molly_franke@hms.harvard.edu)

641 Huntington Avenue, Boston, MA 02118

Supplementary Table 1. Survey response frequency before exposure to social media content by study arm.

| Would you be willing to accept Juan… | Control  N = 113  n (%) | Arm 1  N = 114  n (%) | Arm 2  N = 113  n (%) | Arm 3  N = 114  n (%) | Arm 4  N = 113  n (%) |
| --- | --- | --- | --- | --- | --- |
| …as a foreigner visiting Peru?  Definitely willing  Probably willing  Probably unwilling  Unwilling | 68 (60.2)  39 (34.5)  5 (4.4)  1 (0.9) | 68 (59.7)  35 (30.7)  9 (7.9)  2 (1.8) | 78 (69.0)  31 (27.4)  4 (3.5)  0 (0) | 76 (66.7)  34 (29.8)  3 (2.6)  1 (0.9) | 68 (60.2)  37 (32.7)  5 (4.4)  3 (2.7) |
| …as a citizen in Peru?  Definitely willing  Probably willing  Probably unwilling  Unwilling | 72 (63.7)  37 (32.7)  3 (2.7)  1 (0.9) | 70 (61.4)  36 (31.6)  6 (5.3)  2 (1.8) | 80 (70.8)  29 (25.7)  3 (2.7)  1 (0.9) | 86 (75.4)  27 (23.7)  1 (0.9)  0 (0) | 67 (59.3)  38 (33.6)  6 (5.3)  2 (1.8) |
| …as a coworker?  Definitely willing  Probably willing  Probably unwilling  Unwilling | 65 (57.5)  39 (34.5)  9 (8.0)  0 (0) | 76 (66.7)  31 (27.2)  6 (5.3)  1 (0.9) | 79 (69.9)  32 (28.3)  1 (0.9)  1 (0.9) | 80 (70.2)  31 (27.2)  3 (2.6)  0 (0) | 66 (58.4)  39 (34.5)  5 (4.4)  3 (2.7) |
| …as a neighbor?  Definitely willing  Probably willing  Probably unwilling  Unwilling | 65 (57.5)  39 (34.5)  8 (7.1)  1 (0.9) | 73 (64.0)  35 (30.7)  6 (5.3)  0 (0) | 80 (70.8)  31 (27.4)  1 (0.9)  1 (0.9) | 79 (69.3)  32 (28.1)  3 (2.6)  0 (0) | 64 (56.6)  40 (35.4)  4 (3.5)  5 (4.4) |
| …as a close friend?  Definitely willing  Probably willing  Probably unwilling  Unwilling | 62 (54.9)  38 (33.6)  10 (8.9)  3 (2.7) | 63 (55.3)  39 (34.2)  8 (7.0)  4 (3.5) | 67 (59.3)  41 (36.3)  4 (3.5)  1 (0.9) | 64 (56.1)  43 (37.7)  7 (6.1)  0 (0) | 59 (52.2)  38 (33.6)  10 (8.9)  6 (5.3) |
| …as a partner’s close relative or close relative’s partner?  Definitely willing  Probably willing  Probably unwilling  Unwilling | 45 (39.8)  45 (39.8)  14 (12.4)  9 (8.0) | 49 (43.0)  43 (37.7)  14 (12.3)  8 (7.0) | 55 (48.7)  40 (35.4)  11 (9.7)  7 (6.2) | 50 (43.9)  43 (37.7)  17 (14.9)  4 (3.5) | 51 (45.1)  38 (33.6)  15 (13.3)  9 (8.0) |

Supplementary Table 2. Survey response frequency after exposure to social media content by study arm.

| Would you be willing to accept Juan… | Control  N = 113  n (%) | Arm 1  N = 114  n (%) | Arm 2  N = 113  n (%) | Arm 3  N = 114  n (%) | Arm 4  N = 113  n (%) |
| --- | --- | --- | --- | --- | --- |
| …as a foreigner visiting Peru?  Definitely willing  Probably willing  Probably unwilling  Unwilling | 73 (64.6)  34 (30.1)  6 (5.3)  0 (0) | 84 (73.7)  29 (25.4)  0 (0)  1 (0.9) | 88 (77.9)  21 (18.6)  4 (3.5)  0 (0) | 90 (79.0)  21 (18.4)  3 (2.6)  0 (0) | 76 (67.3)  32 (28.3)  3 (2.7)  2 (1.8) |
| …as a citizen in Peru?  Definitely willing  Probably willing  Probably unwilling  Unwilling | 73 (64.6)  34 (30.1)  6 (5.3)  0 (0) | 76 (66.7)  36 (31.6)  0 (0)  2 (1.8) | 89 (78.8)  21 (18.6)  3 (2.7)  0 (0) | 94 (82.6)  18 (15.8)  1 (0.9)  1 (0.9) | 75 (66.4)  35 (31.0)  2 (1.8)  1 (0.9) |
| …as a coworker?  Definitely willing  Probably willing  Probably unwilling  Unwilling | 71 (62.8)  33 (29.2)  8 (7.1)  1 (0.9) | 81 (71.1)  30 (2.6)  1 (0.9)  2 (1.8) | 88 (77.9)  22 (19.5)  2 (1.8)  1 (0.9) | 91 (79.8)  21 (18.2)  1 (0.9)  1 (0.9) | 75 (66.4)  33 (29.2)  4 (3.5)  1 (0.9) |
| …as a neighbor?  Definitely willing  Probably willing  Probably unwilling  Unwilling | 70 (62.0)  36 (31.9)  7 (6.2)  0 (0) | 82 (71.9)  27 (23.7)  4 (3.5)  1 (0.9) | 89 (78.8)  22 (19.5)  2 (1.8)  0 (0) | 89 (78.1)  21 (18.4)  3 (2.6)  1 (0.9) | 73 (64.6)  34 (30.1)  5 (4.4)  1 (0.9) |
| …as a close friend?  Definitely willing  Probably willing  Probably unwilling  Unwilling | 61 (54.0)  36 (31.9)  13 (11.5)  3 (2.7) | 76 (66.7)  30 (26.3)  6 (5.3)  2 (1.8) | 80 (70.8)  27 (23.9)  4 (3.5)  2 (1.8) | 81 (71.1)  27 (23.7)  4 (3.5)  2 (1.8) | 68 (60.2)  34 (30.1)  9 (8.0)  2 (1.8) |
| …as a partner’s close relative or close relative’s partner?  Definitely willing  Probably willing  Probably unwilling  Unwilling | 46 (40.7)  44 (38.9)  15 (13.3)  8 (7.1) | 64 (56.1)  37 (32.5)  9 (7.9)  4 (3.5) | 71 (62.8)  27 (23.9)  8 (7.1)  7 (6.2) | 68 (59.7)  33 (29.0)  10 (8.8)  3 (2.6) | 61 (54.0)  37 (32.7)  11 (9.7)  4 (3.5) |

Supplementary Table 3. Survey response frequency before and after exposure to social media content by study arm.

| Would you be willing to accept Juan… | Control  N = 113 | | Arm 1  N = 114 | | Arm 2  N = 113 | | Arm 3  N = 114 | | Arm 4  N = 113 | |
| --- | --- | --- | --- | --- | --- | --- | --- | --- | --- | --- |
|  | Pre n (%) | Post n (%) | Pre n (%) | Post n (%) | Pre n (%) | Post n (%) | Pre n (%) | Post n (%) | Pre n (%) | Post n (%) |
| …as a foreigner visiting Peru?  Definitely willing  Probably willing  Probably unwilling  Unwilling | 68 (60.2)  39 (34.5)  5 (4.4)  1 (0.9) | 73 (64.6)  34 (30.1)  6 (5.3)  0 (0) | 68 (59.6)  35 (30.7)  9 (7.9)  2 (1.8) | 84 (73.7)  29 (25.4)  0 (0)  1 (0.9) | 78 (69.0)  31 (27.4)  4 (3.5)  0 (0) | 88 (77.9)  21 (18.6)  4 (3.5)  0 (0) | 76 (66.7)  34 (29.8)  3 (2.6)  1 (0.9) | 90 (79.0)  21 (18.4)  3 (2.6)  0 (0) | 68 (60.2)  37 (32.7)  5 (4.4)  3 (2.6) | 76 (67.3)  32 (28.3)  3 (2.6)  2 (1.8) |
| …as a citizen in Peru?  Definitely willing  Probably willing  Probably unwilling  Unwilling | 72 (63.7)  37 (32.7)  3 (2.6)  1 (0.9) | 73 (64.6)  34 (30.1)  6 (5.3)  0 (0) | 70 (61.4)  36 (31.6)  6 (5.3)  2 (1.8) | 76 (66.7)  36 (31.6)  0 (0)  2 (1.8) | 80 (70.8)  29 (25.7)  3 (2.7)  1 (0.9) | 89 (78.8)  21 (18.6)  3 (2.7)  0 (0) | 86 (75.4)  27 (23.7)  1 (0.9)  0 (0) | 94 (82.6)  18 (15.8)  1 (0.9)  1 (0.9) | 67 (59.3)  38 (33.6)  6 (5.3)  2 (1.8) | 75 (66.4)  35 (31.0)  2 (1.8)  1 (0.9) |
| …as a coworker?  Definitely willing  Probably willing  Probably unwilling  Unwilling | 65 (57.5)  39 (34.5)  9 (8.0)  0 (0) | 71 (62.8)  33 (29.2)  8 (7.1)  1 (0.9) | 76 (66.7)  31 (27.2)  6 (5.3)  1 (0.9) | 81 (71.1)  30 (2.6)  1 (0.9)  2 (1.8) | 79 (69.9)  32 (28.3)  1 (0.9)  1 (0.9) | 88 (77.9)  22 (19.5)  2 (1.8)  1 (0.9) | 80 (70.2)  31 (27.2)  3 (2.6)  0 (0) | 91 (79.8)  21 (18.4)  1 (0.9)  1 (0.9) | 66 (58.4)  39 (34.5)  5 (4.4)  3 (2.7) | 75 (66.4)  33 (29.2)  4 (3.5)  1 (0.9) |
| …as a neighbor?  Definitely willing  Probably willing  Probably unwilling  Unwilling | 65 (57.5)  39 (34.5)  8 (7.1)  1 (0.9) | 70 (62.0)  36 (31.9)  7 (6.2)  0 (0) | 73 (64.0)  35 (30.7)  6 (5.3)  0 (0) | 82 (71.9)  27 (23.7)  4 (3.5)  1 (0.9) | 80 (70.8)  31 (27.4)  1 (0.9)  1 (0.9) | 89 (78.8)  22 (19.5)  2 (1.8)  0 (0) | 79 (69.3)  32 (28.1)  3 (2.6)  0 (0) | 89 (78.1)  21 (18.4)  3 (2.6)  1 (0.9) | 64 (56.6)  40 (35.4)  4 (3.5)  5 (4.4) | 73 (64.6)  34 (30.1)  5 (4.4)  1 (0.9) |
| …as a close friend?  Definitely willing  Probably willing  Probably unwilling  Unwilling | 62 (54.9)  38 (33.6)  10 (8.9)  3 (2.7) | 61 (54.0)  36 (31.9)  13 (11.5)  3 (2.7) | 63 (55.3)  39 (34.2)  8 (7.0)  4 (3.5) | 76 (66.7)  30 (26.3)  6 (5.3)  2 (1.8) | 67 (59.3)  41 (36.3)  4 (3.5)  1 (0.9) | 80 (70.8)  27 (23.9)  4 (3.5)  2 (1.8) | 64 (56.1)  43 (37.7)  7 (6.1)  0 (0) | 81 (71.1)  27 (23.7)  4 (3.5)  2 (1.8) | 59 (52.2)  38 (33.6)  10 (8.9)  6 (5.3) | 68 (60.2)  34 (30.0)  9 (8.0)  2 (1.8) |
| …as a partner’s close relative or close relative’s partner?  Definitely willing  Probably willing  Probably unwilling  Unwilling | 45 (39.8)  45 (39.8)  14 (12.3)  9 (8.0) | 46 (40.7)  44 (38.9)  15 (13.3)  8 (7.1) | 49 (43.0)  43 (37.7)  14 (12.3)  8 (7.0) | 64 (56.1)  37 (32.5)  9 (7.9)  4 (3.5) | 55 (48.7)  40 (35.4)  11 (9.7)  7 (6.2) | 71 (62.8)  27 (23.9)  8 (7.1)  7 (6.2) | 50 (43.9)  43 (37.7)  17 (14.9)  4 (3.5) | 68 (59.7)  33 (29.0)  10 (8.8)  3 (2.6) | 51 (45.1)  38 (33.6)  15 (13.3)  9 (8.0) | 61 (54.0)  37 (32.7)  11 (9.7)  4 (3.5) |

Supplementary Table 4. Median Bogardus change by study arm (original scoring)

|  | Median [5^th^, 95^th^ percentile] | p-value* |
| --- | --- | --- |
| Control | 0 [-1, 2] | - |
| Arm 1 | 0 [-6, 0] | 0.001 |
| Arm 2 | 0 [-1, 0] | 0.08 |
| Arm 3 | 0 [-2, 0] | 0.01 |
| Arm 4 | 0 [-3, 0] | 0.01 |

*P-values from two-sided Wilcoxon rank-sum tests comparing the change in each intervention arm with the change in the control arm.

Supplementary Table 5. Median Bogardus iScore change by study arm

|  | Median [5^th^-95^th^ percentile] | p-value* |
| --- | --- | --- |
| Control | 0 [-10-6] | - |
| Arm 1 | 0 [-16-2] | 0.01 |
| Arm 2 | 0 [-18-2] | 0.16 |
| Arm 3 | 0 [-18-2] | 0.01 |
| Arm 4 | 0 [-17-2] | 0.05 |

*P-values from two-sided Wilcoxon rank-sum tests comparing the change in each intervention arm with the change in the control arm.
